# Microneedles for ophthalmic drug delivery: recent developments

**DOI:** 10.1097/JS9.0000000000000133

**Published:** 2023-03-03

**Authors:** Hitesh Chopra, Priyanka, Om Prakash Choudhary, Talha B. Emran

**Affiliations:** a Chitkara College of Pharmacy, Chitkara University; b Department of Veterinary Microbiology, College of Veterinary Science, Guru Angad Dev Veterinary and Animal Sciences University (GADVASU), Rampura Phul, Bathinda, Punjab; c Department of Veterinary Anatomy and Histology, College of Veterinary Sciences and Animal Husbandry, Central Agricultural University (I), Selesih, Aizawl, Mizoram, India; d Department of Pharmacy, BGC Trust University Bangladesh, Chittagong, Bangladesh; e Department of Pharmacy, Faculty of Allied Health Sciences, Daffodil International University, Dhaka, Bangladesh

*Dear Editor*,

Formulators and retinal specialists have found it difficult to deliver drugs to the eye. Especially for treating disorders that affect hard-to-reach tissues, such as those that affect the posterior segment (PS) of the eye. Age-related macular degeneration, diabetic retinopathy, retinitis pigmentosa, and uveitis are PS disorders that, if untreated, typically cause irreversible vision loss and make up the majority of instances of blindness. Similar to glaucoma, uveitis, and corneal neovascularization, illnesses affecting the anterior portion of the eye are difficult to cure^1^. This is caused by the eye’s recessed location and obstructions that prevent medication molecules from passing through. To get clinically meaningful concentrations of the therapy at the target spot within the eye, a variety of physiological obstacles must be overcome. For instance, obstacles including the sclera, choroid, retinal-pigmented epithelium, retina, and outer/inner limiting membrane must be removed to treat disorders of the PS. In addition, medication molecules must penetrate the conjunctiva, cornea, and tear fluid to treat disorders of the anterior portion of the eyes.

The disadvantages of traditional medication delivery for the eye include the requirement for recurrent drug administration, which results in patient noncompliance^2^. Due to these problems, new drug delivery methods are required to offer both prolonged drug action and tailored drug distribution to overcome the limitations of traditional treatment. Injectable formulations are the most effective among innovative drug delivery technologies because they can precisely deliver the required dosage to the appropriate region of the eye. Injections administered intravenously are administered using standard hypodermic needles. These intraocular injections can be administered by a number of different routes, including the subconjuctival, periocular, intravitreal, and intracorneal. Given this, several drawbacks of intraocular injections include their intrusive nature, frequent administration leading to noncompliance, and decreased absorption.

Microneedles (MNs) are devices constructed of metal or polymer with sizes ranging from a few micrometers to 200 μm^3^. MNs have tiny projections, which reduces their degree of intrusion^4^. These MNs are able to selectively target the medications to the required location of action while also overcoming the drawbacks connected with the currently employed traditional delivery techniques. The MNs method is effective enough to speed up percutaneous medication delivery. MNs are beneficial for percutaneous administration across the oral mucosa, gastrointestinal tract, and even the nail in addition to the potential function they have demonstrated in eye care. These tiny needles, which may be used for a variety of applications, are simple to implant into the eye. These are less uncomfortable than conventional hypodermic needles and may be designed to release the medication gradually over a longer length of time. Consequently, wouldn’t need to be repeated administration. Thakur *et al.* 5 formulated MNs for intraocular delivery, using polyvinylpyrrolidone polymer and using fluorescein sodium and fluorescein isothiocyanate–dextrans as model drugs. The in-vitro data showed enhancement in the permeation of macromolecules across the corneal and scleral tissues in comparison to traditional treatment with an aqueous solution and the polymeric material used was found to be compatible to retinal cells.

As MNs based patches are difficult to retain in the eye thus for this Amer and Chen 6 developed MN-based hydrogel patches having self-adhesive properties, when exposed to media, the hydrogels swell and get attached to the inner layer. Lee and colleagues developed rapidly detachable MN pen-based technology that contains the dissolvable sacrificial layer that develops as the polymer oozes out of the tip. The porous layer was fabricated with polyvinyl alcohol and polyvinylpyrrolidone. Due to the presence of porous structures, the dissolution is accelerated due to the capillary effect coming into play. The formulation showed detachment of MN layer and gets embedded into the porcine sclera. This type of formulation is a boon in the case of a person suffering from keratitis or glaucoma^7^. Jung and colleagues described more advanced technologies including the use of iontophoresis that helps to direct the delivery of anionically charged nanoparticles through the suprachoroidal space towards the PS of the eye. In the *ex vivo*, based study it was found that without iontophoresis nanoparticles become localized near the limbus region only, and a fraction of nanoparticles were delivered to the posterior region of suprachondrial space. However, using the iontophoresis, more than 30% of the drug was able to reach the posterior region of suprachondrial space; the findings were concordant with the in-vivo data^8^.

## Future directions

The benefits of using MNs as ocular drug delivery systems are undeniable, but there are also some significant downsides, as was demonstrated in the research that were examined. The literature studies’ materials and methodologies make it clear that the fastest-dissolving systems obtained by using hydrophilic polymers – are the focus of the most significant trends in this field. To reduce discomfort and the chance of adverse effects such as irritation, tissue damage, infection, and inflammation, these formulations are designed for brief residence durations at the eye surface^4^. However, there is currently a lack of information regarding the potential risk associated with ocular MNs, necessitating more research in this field^9^. There are currently no attempts to introduce any MN-based ophthalmic formulations to the pharmaceutical market, except for those containing a single MN rather than MN arrays, as the majority of the safety issues still need to be thoroughly explored. However, the papers reporting single MN injections can teach us some crucial things. The patient may experience the formulation as painful or tolerable depending on a number of factors, such as the volume of liquid injected. As a result, each of these factors needs to be thoroughly evaluated and adjusted. As we have already mentioned, randomized clinical investigations with MN arrays are also necessary.

## Ethical approval

Not applicable.

## Sources of funding

None.

## Authors’ contribution

H.C.: conceptualization, data curation, writing – original draft preparation, writing – reviewing and editing. Priyanka and O.P.C.: data curation, writing – original draft preparation, writing – reviewing and editing. T.B.E.: writing – reviewing and editing, visualization, supervision.

## Conflicts of interest disclosure

The authors declare that they have no financial conflict of interest with regard to the content of this report.

## Research registration unique identifying number (UIN)

Not applicable.

## Guarantor

Talha B. Emran.

## Provenance and peer review

Not commissioned, internally peer-reviewed.
